# Telephone Outreach Enhances Recruitment of Underrepresented Seriously Ill Patients for an Advance Care Planning Pragmatic Trial

**DOI:** 10.1007/s11606-022-08000-7

**Published:** 2023-01-30

**Authors:** Aaron J. Chau, Rebecca L. Sudore, Ron D. Hays, Chi-Hong Tseng, Anne M. Walling, Maryam Rahimi, Lisa Gibbs, Kanan Patel, Fernando Javier Sanz Vidorreta, Neil S. Wenger

**Affiliations:** 1 Division of Geriatric Medicine, Department of Family Medicine, University of California, Irvine School of Medicine, Irvine, CA USA; 2 Department of Medicine, University of California, San Francisco School of Medicine, San Francisco, CA USA; 3 San Francisco Veterans Affairs Health Care System, San Francisco, CA, USA; 4 UCLA Division of General Internal Medicine and Health Services Research, University of California, Los Angeles School of Medicine, Los Angeles, CA USA; 5 VA Greater Los Angeles Health System, Los Angeles, CA USA; 6 Department of Medicine, University of California, Irvine School of Medicine, Irvine, CA USA

**Keywords:** RDS: vulnerable populations, disparities, recruitment, health research, phone calls, advance care planning

## Abstract

**Background:**

Patients experiencing systemic patterns of disadvantage, such as racial/ethnic minorities and those with limited English proficiency, are underrepresented in research. This is particularly true for large pragmatic trials of potentially sensitive research topics, such as advance care planning (ACP). It is unclear how phone outreach may affect research participation by underrepresented individuals.

**Objective:**

To assess the effect of phone outreach, in addition to standard mail survey recruitment, in a population-based ACP pragmatic trial at three academic health systems in California.

**Design:**

Retrospective cohort study

**Patients:**

Primary care patients with serious illness were mailed a survey in their preferred language. Patients who did not initially respond by mail received up to three reminder phone calls with the option of survey completion by phone.

**Main measures:**

Effect of phone outreach on survey response rate associated with respondent demographic characteristics (e.g., Social Vulnerability Index [SVI], range 0 (low) to 1 (high)).

**Results:**

Across the health systems, 5998 seriously ill patients were mailed surveys. We obtained completed surveys from 1215 patients (20% response rate); 787 (65%) responded after mail alone and 428 (35%) participated only after phone outreach. Patients recruited after phone outreach compared to mail alone were more socially vulnerable (SVI 0.41 v 0.35, *P* < 0.001), were more likely to report being a racial/ethnic minority (35% v 28%, *P* = 0.006), and non-English speaking (16% v 10%, *P* = 0.005). Age and gender did not differ significantly. The inclusion of phone outreach resulted in a sample that better represented the baseline population than mail alone in racial/ethnic minority (28% mail alone, 30% including phone outreach, 36% baseline population), non-English language preference (10%, 12%, 15%, respectively), and SVI (0.35, 0.37, 0.38, respectively).

**Conclusions:**

Phone outreach for a population-based survey in a pragmatic trial concerning a potentially sensitive topic significantly enhanced recruitment of underrepresented seriously ill patients.

## INTRODUCTION

Patients experiencing disadvantage, such as the socioeconomically disadvantaged, seriously ill, racial/ethnic minorities, and those with limited English proficiency, are historically underrepresented in research, especially concerning potentially sensitive topics such as advance care planning (ACP).^1,2^ The ability to generalize research findings, particularly to underrepresented subgroups, is limited when participants in a sample do not reflect the target population.^3^ These knowledge gaps hinder efforts to address health disparities that persist for underrepresented patients.^4,5^ Despite mandates to include historically underrepresented populations, it remains a challenge for researchers to recruit representative samples, particularly for large pragmatic trials.^6^ Documenting techniques that improve the recruitment of underrepresented patients is needed.

Many clinical research studies do not recruit their target sample size.^7^ To improve recruitment and representation, a variety of techniques can be used. Mailing, phone calls, media marketing, and community outreach enhance research participation and sample diversity with varying degrees of success.^8^ In clinical trials and population-based survey studies, mail and phone reminders increase study participation, with phone reminders being more effective.^9,10^ However, it is unknown whether phone outreach enhances the recruitment of underrepresented patients, particularly for large pragmatic trials and research on sensitive topics, such as ACP.

Therefore, we assessed the recruitment efforts of seriously ill primary care patients for a population-based ACP pragmatic trial at three academic health systems in California.^11^ Because underrepresented groups are less likely to engage in ACP, it was critical to enroll and survey a diverse sample of individuals that included historically underrepresented patients.^12^ We examined the effect of phone outreach in addition to standard mail recruitment on the inclusion of underrepresented patients in the research sample and whether it better reflected the diversity of the baseline patient population of seriously ill patients compared to mail alone.

## METHODS

We analyzed response rates to a survey distributed to all seriously ill primary care patients at three University of California health systems and the characteristics of respondents. As part of a pragmatic ACP trial, ACP mailings and electronic health record (EHR) messages were sent out to every patient 18 years and older identified as having serious illness based on a medical record–validated EHR algorithm and lacking an advance directive or Physician Orders for Life Sustaining Treatment (POLST) documented in the EHR within the past 3 years.^11^ In addition to the population-based rollout of the ACP intervention, the project aimed to survey a representative sample of this seriously ill population to understand attitudes toward and readiness to participate in ACP, care preferences, reports about communication by providers, and health status. This study was approved by the Institutional Review Board at UCLA (18-001612) with delegation from the other two health systems.

English- and Spanish-language-preference patients were mailed a survey in their preferred language. Across the three health systems, surveys were mailed in the latter half of 2019 and beginning of 2020. Patients who did not respond to the mailed survey were called by staff up to three times to remind them of the mailed survey or to help complete the survey over the phone. Patients were called in order of ascending medical record number at each site. Because the number of patients not returning surveys exceeded the available research assistant time for phone calls, not every patient received a phone call. After recruitment was completed, we examined the demographic characteristics and social vulnerability of patients who were recruited with and without phone outreach.

Research staff recorded the dates and outcomes of survey mailing and phone outreach. They also recorded dates and reasons for patient refusal, and dates and methods of survey completion. We developed computer code to identify the timing of logged phone calls across the three sites and merged these with survey outcome data.

Patient demographic characteristics for the baseline seriously ill primary care population and demographics of the individuals recruited by mail alone and by phone were obtained from the EHR at each health system. These included gender, race, ethnicity, preferred language, home street address, and date of birth. Age was calculated at the date that the survey was mailed to the patient.

We measured social vulnerability using the Social Vulnerability Index (SVI), which is based on 15 US Census variables, such as income, educational level, employment, crowding, and vehicle access.^13,14^ SVI is an indicator of potential negative effects from external stress on health. An SVI score is attributed to each census tract ranging from 0 to 1, with 1 being the most vulnerable. Patient home addresses in our population were geocoded using ArcGIS Pro to obtain geographical coordinates. The coordinates were then binned into census tracts, which allowed SVI scores to be matched to each patient.

### Statistical Analysis

Patients were categorized as recruited by mail alone if they completed the survey without the need for a call. Patients were categorized as recruited after phone outreach if a call was made before the survey was completed. Phone calls were considered valid if the call resulted in either verbal contact with the patient or a voicemail message.

We compared demographic characteristics, including SVI, between patients recruited after mail alone and after phone outreach using chi-square tests for categorical variables and *t* tests for continuous variables. We coded racial/ethnic minority as Hispanic/Latinx, Black/African American, Native Hawaiian/other Pacific Islander, American Indian/Alaska Native, and other race/ethnicity.

The outcomes of phone calls where verbal contact was made with patients were displayed graphically by time and day of week. Phone calls resulting in voicemails, busy lines, or other non-contact outcomes were not included in the graphical analysis. Verbal contact outcomes were categorized into “completed survey” if the patient completed the survey either during the phone call with research staff or by mail after the phone call; “opted out” if patients actively opted out of the survey during the phone call with staff or by leaving a voicemail after the phone call; and “other (no survey)” if patients were contacted by phone, agreed to consider completing the survey, but never completed the survey.

We used descriptive statistics, and associations between participation and demographic variables were assessed with chi-square and *t* tests. Analyses were performed using Python version 3.9.1.

## RESULTS

Across the three academic health systems, 5998 seriously ill patients were mailed surveys. The mean age of the population of seriously ill patients eligible for survey was 71 years and mean SVI was 0.38. The patients were 52% male, 85% English speaking, 53% White, 19% Hispanic/Latinx, 11% Asian/Pacific Islander, 9% Black/African American, and 9% other race/ethnicity. We obtained completed surveys from 1215 patients (20% response rate). The mean age of survey respondents was 69 years and mean SVI was 0.37. Responding patients were 52% male, 88% English speaking, 61% White, 18% Hispanic/Latinx, 9% Asian/Pacific Islander, 7% Black/African American, and 5% other race/ethnicity (Table 1).

**Table 1:** Comparison of Characteristics of Patients Who Completed Surveys After Mail Alone Vs After Mail and Then Phone Outreach, and Description of Patients in Resultant Full Survey Sample and Total Population of Seriously Ill Patients

	Surveys completed		Overall	
	Mail alone (*N* = 787)	Mail and then phone outreach (*N* = 428)	Full survey sample (*N* = 1215)	Population of seriously ill patients (*N* = 5998)
Age, mean (SD)	69.6 (15.7)	69.0 (14.5)	69.4 (15.3)	71.3 (14.6)
Gender, *N* (%)				
Male	415 (52.7)	217 (50.7)	632 (52.0)	3120 (52.0)
Female	370 (47.0)	209 (48.8)	579 (47.7)	2873 (47.9)
Non-binary	2 (0.3)	2 (0.5)	4 (0.3)	4 (0.1)
Race/ethnicity, *N* (%)*				
White	502 (63.8)	238 (55.6)	740 (60.9)	3171 (52.9)
Hispanic or Latinx	132 (16.8)	82 (19.2)	214 (17.6)	1108 (18.5)
Asian	67 (8.5)	39 (9.1)	106 (8.7)	681 (11.4)
Black or African American	46 (5.8)	39 (9.1)	85 (7.0)	527 (8.8)
Native Hawaiian, Pacific Islander	2 (0.3)	4 (0.9)	6 (0.5)	23 (0.4)
American Indian, Alaska Native	0 (0)	2 (0.5)	2 (0.2)	14 (0.2)
Other	38 (4.8)	24 (5.6)	62 (5.1)	474 (7.9)
Language, *N* (%)^†^				
English	706 (89.7)	360 (84.1)	1066 (87.7)	5120 (85.4)
Spanish	70 (8.9)	55 (12.9)	125 (10.3)	574 (9.6)
Other	11 (1.4)	13 (3.0)	24 (2.0)	304 (5.1)
SVI, mean (SD)^‡^				
Overall	0.35 (0.27)	0.41 (0.27)	0.37 (0.27)	0.38 (0.27)
Socioeconomic	0.33 (0.25)	0.38 (0.26)	0.35 (0.26)	0.36 (0.26)
Household composition	0.36 (0.25)	0.39 (0.25)	0.37 (0.25)	0.37 (0.26)
Minority and language	0.39 (0.27)	0.45 (0.27)	0.41 (0.27)	0.42 (0.27)
Housing and transportation	0.44 (0.29)	0.51 (0.29)	0.47 (0.29)	0.47 (0.29)

Column 1 contains patients who completed the survey after mail alone. Column 2 contains patients who completed the survey after mail and then phone follow-up. Column 3 combines columns 1 and 2 and is the full survey sample. Column 4 is the population of seriously ill patients eligible to complete the survey

*Chi-square *P* = 0.006 comparing White/Asian to all other race/ethnicities in surveys completed after mail alone versus after mail and then phone outreach (columns 1 and 2)

^†^Chi-square *P* = 0.005 comparing English to all other languages in surveys completed after mail alone versus after mail and then phone outreach (columns 1 and 2)

^‡^*T* test *P* < 0.001 for Social Vulnerability Index (SVI, scores range from 0 to 1, with 1 indicating more vulnerability) in surveys completed after mail alone versus after mail and then phone outreach (columns 1 and 2)

**Fig. 1:**
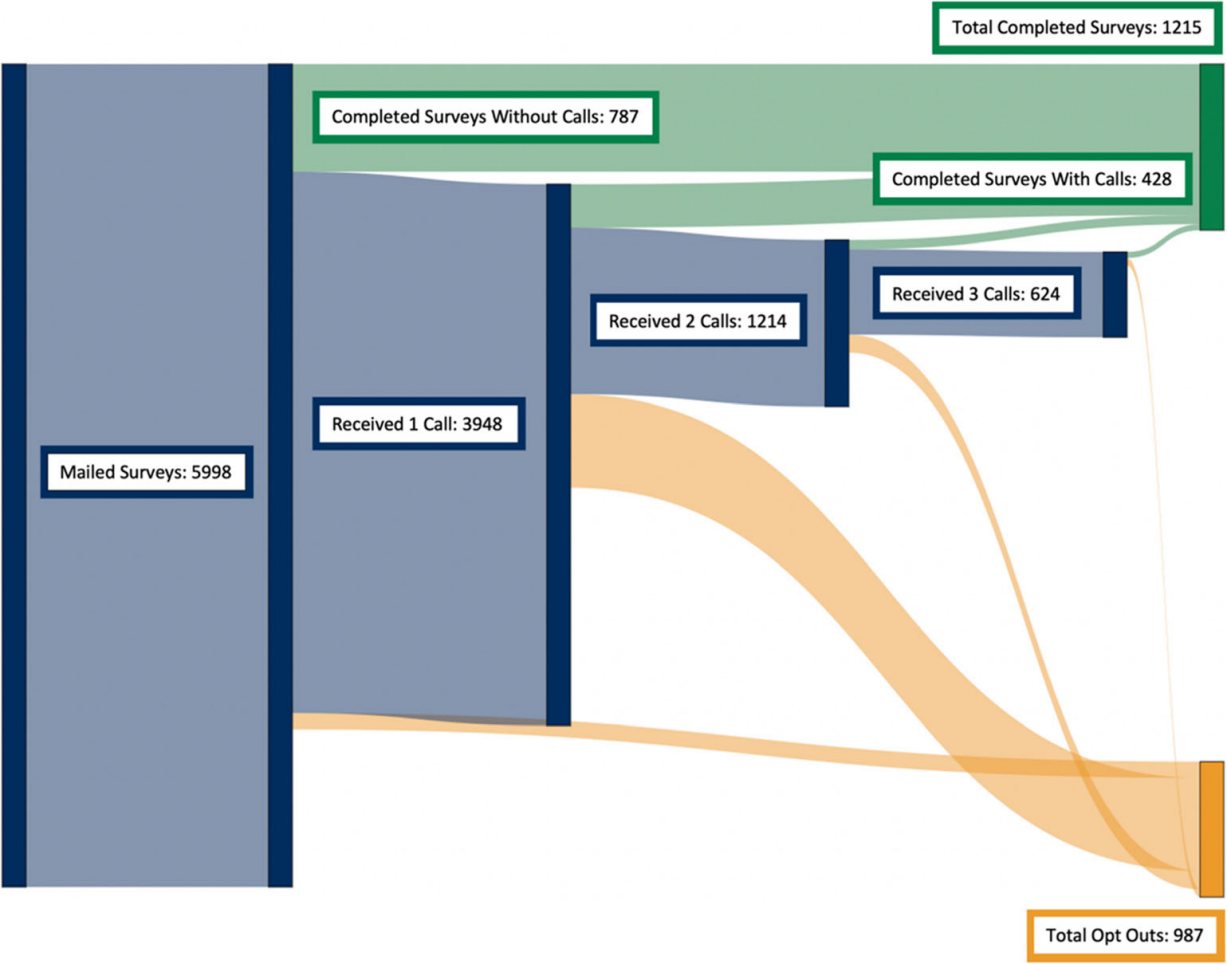
Sankey diagram showing proportion of survey completion, phone outreach, and opt outs among the 5998 seriously ill patients who were mailed surveys.

Of the 1215 seriously ill patients who completed the survey, 787 (13%) did so after mail recruitment alone and 428 (7%) after phone outreach (representing 35% of the total surveys completed; see Fig. 1). Among the 5096 patients who had not completed a survey by mail alone or opted out by mail, 3948 (77%) received a first phone call from staff and 318 additional surveys were obtained. Of the 2949 patients who had not responded to the first phone call or opted out, 1214 (41%) received a second phone call and 66 additional surveys were obtained. Of the 1017 patients who had not responded to the second call or opted out, 624 (61%) received a third phone call and 44 additional surveys were obtained.

Compared to patients recruited by mail alone, patients recruited only after phone outreach were more socially vulnerable (SVI 0.41 phone v 0.35 mail alone, *P* < 0.001), and included a greater proportion of non-White, non-Asian racial/ethnic minorities (35% v 28%, *χ*2 (1 df) = 7.567, *P* = 0.006) and those with a non-English language preference (16% v 10%, χ2 (1 df) = 8.343, *P* = 0.005) (Table 1). There was no statistically significant difference in age or gender.

The inclusion of patients recruited after phone outreach resulted in a sample that better represented the seriously ill survey eligible population in terms of non-White, non-Asian racial/ethnic minorities (28% mail alone, 30% including phone outreach, 36% baseline population), non-English language (10%, 12%, 15%, respectively), and SVI (0.35, 0.37, 0.38, respectively) (Table 1).

**Fig. 2:**
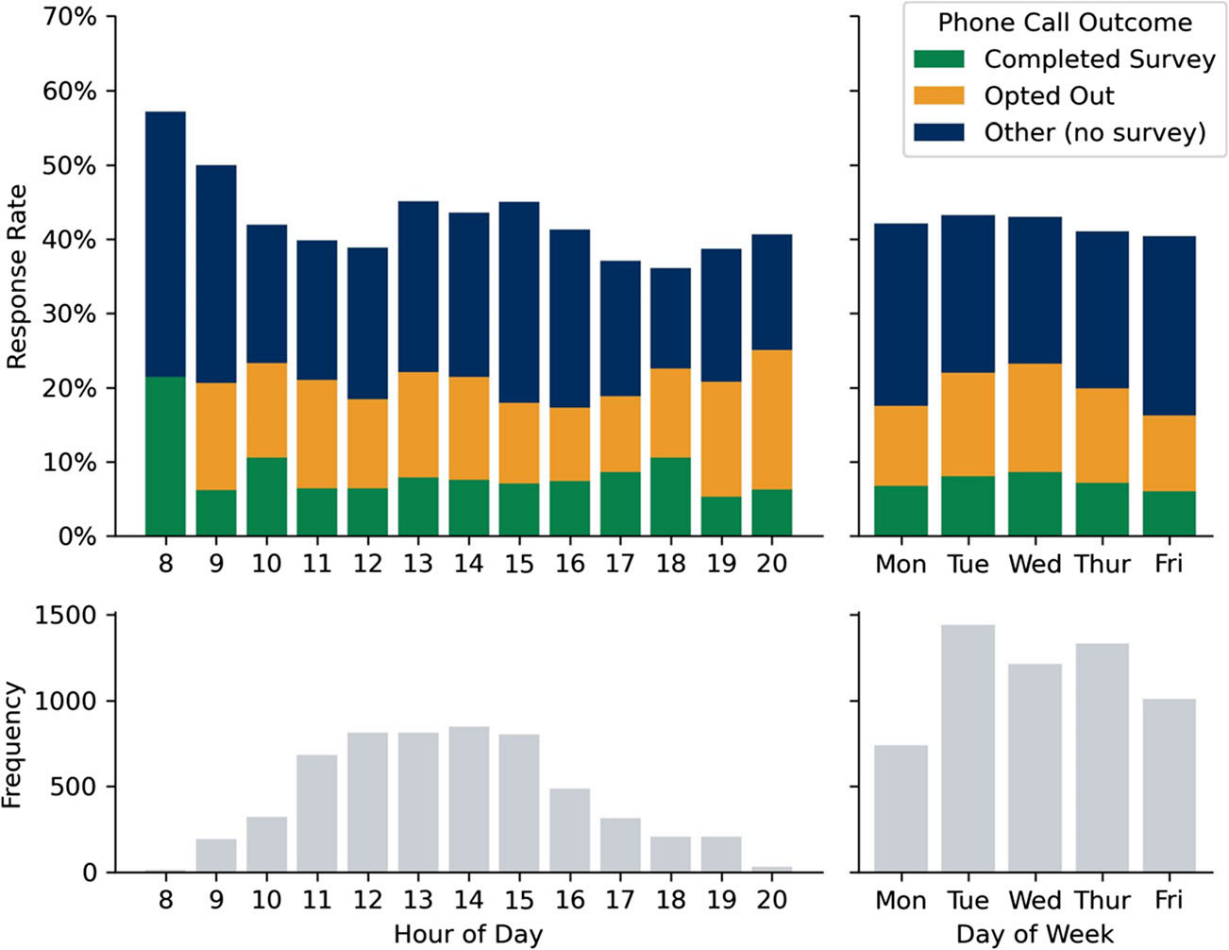
Upper plots show responses to phone calls by time of day and day of week. Note that only telephone calls resulting in verbal contact with the patient are shown. Lower plots show the frequency of all phone calls by time of day and day of week.

In terms of timing and efficacy, phone calls completed between 8 a.m. and 10 a.m., and 1 p.m. and 4 p.m., reached more patients; however, survey completion rates were similar throughout the day. Opt outs appear to be higher for phone calls placed between 7 p.m. and 9 p.m. (Fig. 2). Phone calls were made by research staff between 8 a.m. and 8 p.m. on Monday through Friday.

## DISCUSSION

Recruiting a sample representative of the baseline clinical population is important for the generalizability of research findings. In this multisite pragmatic trial of ACP, we evaluated the effectiveness of our phone outreach to capture a representative research sample. We found that phone outreach after a survey mailing increased participation by more socially vulnerable, racial/ethnic minority, and non-English speaking seriously ill patients. The inclusion of these patients resulted in a research cohort that more closely represented the baseline clinical population of seriously ill primary care patients.

As shown in previous research, survey response rates can vary significantly between demographic groups.^15^ Digital media, such as phone calls, text messages, and email, have been used successfully in the recruitment of underrepresented older adults.^16,17^ Our findings on the effectiveness of phone outreach align with these results. This is a critical step in carrying out population-based research on vulnerable patients such as those who have serious illness. Our findings show that phone outreach not only increases recruitment and enhances diversity, but also can be used for studies that focus on potentially sensitive topics (e.g., ACP). This has implications for the allocation of resources in population-based survey efforts: resources for outreach should be prioritized in order to enhance respondent representativeness. This study also demonstrated that exploring comparisons between enrolled research patients and the baseline clinical population is feasible. Using geocoded data can enhance understanding about whether outreach affects the representativeness of the sample.

In terms of timing of calls, it appears that patients in this analysis were more responsive to phone outreach in the early morning and early afternoon. This could be an area for further research with a more robust prospective data collection process than what was used in this study. In addition, more information would be needed to understand whether the patients in the cohort are still working outside the house and/or have caregiving duties during the day or after hours. We were unable to conduct calls on weekends, which may enhance recruitment or increase the number of opt outs. Our data demonstrate that there appears to be a “bewitching” hour at 7 p.m. as calls made after that time resulted in higher refusal rates.

This study has several limitations. The project was conducted in three academic health systems in California, and these findings may not apply to community or rural health systems. The survey topic of ACP is potentially sensitive, especially for seriously ill patients, and our findings may not apply to research in other areas. The population of patients eligible for the survey was composed of seriously ill patients without ACP documentation in the EHR and as such was more vulnerable (older; more likely to be Black/African American, Hispanic/Latinx or other; and higher SVI) than the seriously ill patients who had completed ACP documentation. In addition, there were limitations in phone outreach to our large sample. Because the number of patients not returning surveys exceeded available research assistant time, not every patient received phone call follow-up. Follow-up of additional patients likely would have yielded a higher survey completion rate. This study was not designed to capture the cost of research assistant phone call follow-up. The cost-effectiveness of phone calls versus other mechanisms of improving response, and other methods of enhancing generalizability, such as employing survey non-response weights, should be evaluated in future studies.

In a pragmatic trial concerning the potentially sensitive topic of ACP, we found that phone outreach significantly enhanced the recruitment of underrepresented seriously ill primary care patients. Identifying additional techniques to improve underrepresented patient recruitment is paramount to ensuring inclusion and representativeness and decreasing health disparities.
